# Brain aging and dementia during the transition from late adulthood to old age: design and methodology of the “Invece.Ab” population-based study

**DOI:** 10.1186/1471-2318-13-98

**Published:** 2013-09-24

**Authors:** Antonio Guaita, Mauro Colombo, Roberta Vaccaro, Silvia Fossi, Silvia Francesca Vitali, Gianluigi Forloni, Letizia Polito, Annalisa Davin, Virginia Valeria Ferretti, Simona Villani

**Affiliations:** 1 “Golgi Cenci” Foundation, Corso San Martino 8, 20081 Abbiategrasso, Italy; 2 “Mario Negri” Institute for Pharmacological Research, Via La Masa 19, 20156 Milan, Italy; 3 “C.Golgi” Geriatric Institute, Piazza Golgi 11, 20081 Abbiategrasso, Italy; 4 Biostatistics and Clinical Epidemiology, Department of Public Health, Neurosciences, Experimental and Forensic Medicine, University of Pavia, Via Mondino 2, 27100 Pavia, Italy

**Keywords:** Dementia, Mild cognitive impairment, Alzheimer’s disease, Brain aging, Longitudinal study

## Abstract

**Background:**

Developed countries are experiencing an unprecedented increase in life expectancy that is accompanied by a tremendous rise in the number of people with dementia. The purpose of this paper is to report on the study design and methodology of an Italian population-based study on brain aging and dementia in the elderly. This multi-domain study is structured in two phases. Our goal is to gather sufficient data to estimate the prevalence (phase I: cross-sectional study), the incidence and the progression of dementia and its subtypes as well as cognitive impairment (phase II: follow-up study) and to identify socio-demographic, clinical, and lifestyle factors associated with dementia and the quality of brain aging in people aged 70–74 years, a crucial point between late adulthood and old age.

**Methods/Design:**

We chose to contact all 1773 people born between 1935–39 residing in Abbiategrasso, Milan, Italy. Those who agreed to participate in the “Invece.Ab” study were enrolled in a cross-sectional assessment and will be contacted two and four years after the initial data collection to participate in the longitudinal survey. Both the cross-sectional and longitudinal assessments include a medical evaluation, a neuropsychological test battery, several anthropometric measurements, a social and lifestyle interview, blood analyses, and the storage of a blood sample for the evaluation of putative biological markers.

**Discussion:**

Now at the end of the recruitment phase, the evaluable population has amounted to 1644 people. Among these, 1321 (80.35%) of the participants have completed phase I. This high return rate was likely due to the style of recruitment and personalization of the contacts.

**Trial registration:**

NCT01345110

## Background

### Brain aging and dementia: a public health emergency

Brain aging is characterized by numerous physiological, structural, functional and neurocognitive changes with a large variability among the aging population. Aging increases the risk of disabilities such as dementia, i.e., the gradual deterioration of mental function affecting the performance of normal daily activities [1]. The prevalence of dementia in people over sixty five has been estimated between 5.9% [2] and 8% [3] in the Italian population. In European countries, the prevalence of dementia is about 6.4% in people over 65 years, and this percentage doubles every five years after this age [4]. Considering the recent increase in longevity, dementia is expected to affect a large number of people in the foreseeable future. This increase will likely have a dramatic impact on the quality of life of the affected population and on the social costs of caregiving. For this reason, improving the health and well-being of dementia patients is a top priority [5].

The differences in the reported prevalence of dementia, according to the aforementioned study, depend on several factors. One important aspect is that most of the studies that estimated the prevalence of dementia were conducted with cohorts with a wide age range. Once the study population is stratified by age group, the sample size is often not sufficient to reach powerful statistical conclusions. Large multicenter studies or meta-analyses [6] may reduce this problem. However, these studies are inevitably conducted in different geographical areas, thus introducing variability that might impact the interpretation of the data. Another important issue, thought to contribute to prevalence estimate heterogeneity, is that the majority of the studies, at least in Europe, adopts a two-phase approach, which consists of a screening phase, generally administering MMSE, followed by a diagnostic phase that include clinical and neuropsychological examination [7].

To overcome these limitations, a population-based study (“Invece.Ab” **Inve**cchiamento **Ce**rebrale in **Ab**biategrasso, i.e., Brain aging in Abbiategrasso), coordinated by the Golgi Cenci Foundation (GCF), was conducted in people living in Abbiategrasso (Milan, Italy) who were born between 1935 and 1939. This study consisted on a multi-dimensional assessment including a clinical and neuropsychological examination extended to the whole population in a single-phase fashion.

In the past 30 years, prospective population studies on dementia have highlighted multiple risk factors besides aging (e.g., family history of dementia years of education). Lifestyle changes (e.g., physical and social inactivity, diet, alcohol consumption, or smoking) that can reduce risk, even in late adulthood, are very useful in developing preventive strategies. In this regard, multidimensional observational approaches are useful to depict an exhaustive risk profile of unsuccessful brain aging.

### Risk factors for dementia

#### Socio-demographic factors

Educational level has repeatedly been reported to have an inverse relationship with dementia [8]; while there is less evidence to demonstrate the influence of occupation [9].

#### Clinical risk factors

Vascular risk factors (heart disease, stroke, hypertension, central obesity, diabetes, and elevated plasma homocysteine and cholesterol concentrations) are known to increase the risk of dementia and accelerate the associated cognitive decline [10-15]. In addition, apolipoprotein E (ApoE) is currently considered to be an important genetic marker for late onset Alzheimer’s disease. The ϵ4 allele of the ApoE gene is associated with an increased risk of both familial and sporadic forms of Alzheimer’s, accounting for 20–50% of the attributable risk [16,17].

#### Anthropometrics measures

Both a low and high BMI are considered to be risk factors for dementia [18], whereas the influence of overweight health status and metabolic syndrome are still up for debate [19,20]. A smaller head circumference has been associated with an increased risk of developing Alzheimer's disease [21]. Moreover, AD patients with a larger head circumference perform better on cognitive tasks than patients with a smaller head circumference who have similar brain pathology [22].

#### Environmental and lifestyle factors

Participation in cognitively stimulating activities and the maintenance of an active and socially integrated lifestyle have both been found to delay the onset of dementia [23-25]. In addition, consuming relatively high levels of vegetables and fish (n-3 polyunsaturated fatty acids) has been associated with a reduced risk of incident Alzheimer’s disease [26]. The moderate consumption of alcohol has been observed to be a protective factor in non-ApoE ϵ4 carriers, whereas heavy drinking may accelerate the onset of Alzheimer’s disease [27]. Caffeine consumption has been associated with a reduction in the number of white matter lesions and potentially the delayed onset of dementia [28]. In previous research, nicotine intake had been associated with a decreased risk of dementia [29], but a more recent meta-analysis found the results produced by case–control and cohort studies to be inconclusive with respect to the direction of this relationship [30]. Sleep disorders appear to be frequent among those with early dementia, although self-reported sleep difficulties in late adulthood are questionable as predictors of cognitive decline [31]. Despite the clear relevance, there exist few prospective studies of the relationship between sleep disorders and dementia. Furthermore, the feeling of being older than one’s actual age is a predictor of poor general health. In particular, higher perceived age is associated with increased mortality [32]. Mental stress associated with one’s primary occupation is thought to influence cognitive performance in late adult life. Adverse life events are well known risk factors for depression [33]. Considering the role of mood in the onset of dementia, increased stress associated with negative life events may also be a risk factor for dementia [34].

Engaging with technological devices such as cell phones, computers, and remote controls has been correlated with activation of neuropsychological systems, and therefore the cognitive profile can also have an effect on everyday use of technology [35].

### Aims of the “Invece.Ab” study

The goals of the “InveCe.Ab” study are as follows:

1) Estimate the prevalence and incidence of dementia and its subtypes, mild cognitive impairment (MCI), and cognitive impairment with no dementia (CIND) in people 70–74 years of age at baseline;

2) Investigate the potential risk or protective value of various epidemiologic, clinical, and biological factors as determinants of the quality of cognitive aging and dementia onset. Special attention is given to understanding how modifiable risk factors impact cognitive impairment with and without dementia. In this paper we describe the study design and methods, together with the preliminary recruitment results of the “Invece.Ab” study.

## Methods/Design

### Study design

The Invece.Ab study is structured in two phases:

1) Cross-sectional study. The aims of this sub-study were to investigate the prevalence of dementia with subtypes as well as the rate of cognitive impairment and to investigate the influence of several potential risk factors in our target population. In this regard, we used biological, social, clinical, and neuropsychological measures to collect data from our participants.

2) Follow-up study. After 2 and 4 years each participant will be re-examined using procedures that are identical to those used in the cross-sectional study. The primary goal of the longitudinal evaluation is to establish the incidence of dementia and its subtypes, as well as the level of cognitive impairment. In addition, we wish to assess whether the investigated parameters have predictive value with respect to the quality of brain aging as well as dementia onset and\or progression.

### Study area and participants

**Figure 1:**
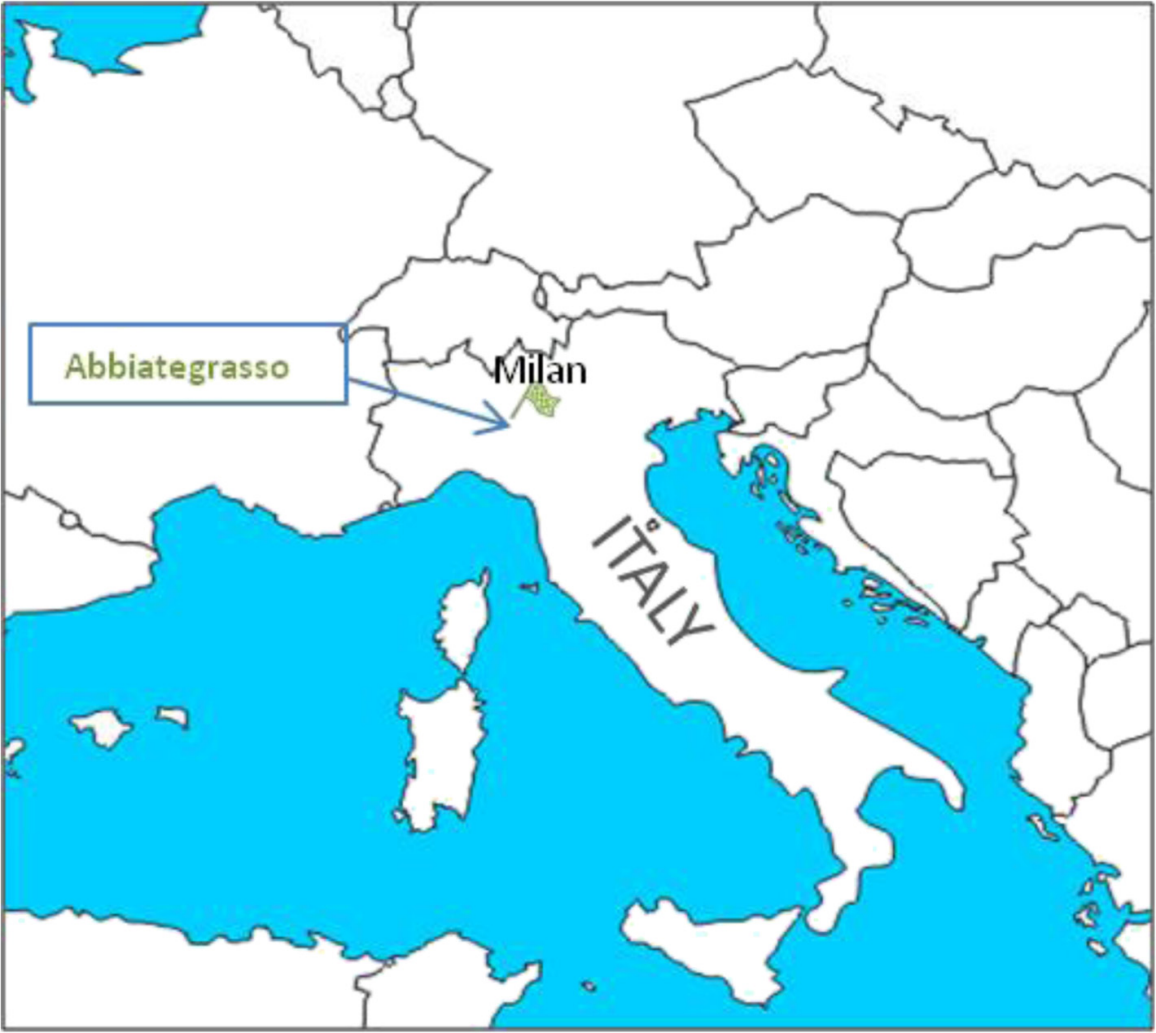
Map of the area of the “InveCe.Ab” study.

The “Invece.Ab” study is set in Abbiategrasso, a small city in the province of Milan (North Italy; see Figure 1) with 30,000 inhabitants. The eligible population comprises all people born between 1935 and 1939 who were residents living in Abbiategrasso on the start date of the study (1.773). This included people who were institutionalized.

Following a single age quintile in a longitudinal study format allows us to minimize the confounding effect of age. The study is being conducted in a specific geographic area; hence, the recruited cohort is homogeneous with respect to age and ethnicity. Moreover, the choice to study individuals aged 70–74 years is based on the observation that this is considered to be a "transitional age" between late adulthood and old age, especially in terms of cognitive function [36], and that social and lifestyle factors still influence cognitive aging during this time [36].

### Recruitment

A list of eligible people was obtained from the municipal registry office. Prior to conducting follow-up measures, this list will be updated for relevant changes (e.g. hospitalizations, deaths, and others) with the help of local health authorities, municipality, hospitals and general practitioners.

The study procedures were in accordance with the principles outlined in the Declaration of Helsinki of 1964 and the following amendments. The study protocol was submitted to and approved by the Ethics Committee of the University of Pavia. Each phase of research was under the supervision of the “Federazione Alzheimer Italia”, the key association supporting those affected by dementia and their relatives in Italy.

Recruitment was conducted with the involvement of local social networks and voluntary associations [37]. The design and aims of the survey were described in local newspapers and discussed in meetings with general practitioners, parishes, and volunteer organizations. All eligible individuals received a letter inviting them to attend a public presentation about the “Invece.Ab” study. Public presentations about the survey were carried out separately for each cohort by birth-year (1935, 1936, 1937, 1938, 1939). During these meetings the first voluntary candidates were recruited. Those who did not volunteer to participate during the meetings received a letter explaining the study aims and inviting them to participate with a confirmed appointment date. They were also contacted by phone if their phone number was available.

The evaluable population excluded those who were not residing in the study area during the baseline study period, had permanently moved away, were deceased, and those who could not be located despite several phone calls and three letters sent to the address provided by the Municipality. All participants provided written informed consent for the use of personal data and agreed to provide a blood sample that would be used for biological analyses and DNA extraction. For participants with severe cognitive impairments, written informed consent was obtained from their relatives or caregivers.

The longitudinal phase will include all participants who participated to the cross-sectional assessment, excluding those who have since passed away, have moved to another city, or those who are no longer willing to participate to the study.

### Study development

Each participant underwent a comprehensive clinical evaluation that incorporated social, medical and psychological examinations. All assessments were carried out at the GCF with the exception of examinations of participants with significant health problems, which took place at their homes. The assessments were divided into two appointments. The first was about 1.5 hours in duration and included blood sampling, a social questionnaire, and an evaluation of walking speed. The second appointment was about 2 hours in duration and included a medical examination and a neuropsychological assessment of mood and cognitive functions. Following these evaluations each participant received a letter with the results of the hematologic analyses and neuropsychological tests. The first follow-up assessments are scheduled to take place approximately 24 months after the first examination, the second after 48 months. The entire study is expected to run until 2014.

A pilot study was conducted prior to the main experiment. This was necessary to evaluate the feasibility of each instrument (e.g., the medical and social questionnaires and the neuropsychological test batteries) on a sample of people with a similar age and geographic area who were attending social and sanitary services. In addition, a pilot study was useful for training the interviewers responsible for administering the social questionnaires, and allowed us to generate guidelines for compiling the questionnaires in a way that would guarantee homogeneity in the data collection.

At each step of the follow-up, responders will be compared with non-responders with respect to certain crucial characteristics (sex, age, birth region, health, and education) that are available at the registry office of Abbiategrasso, to detect any non-response bias.

### Endpoints and diagnostic criteria

The main anticipated endpoints of the study are dementia syndrome and cognitive impairment. Each participant will receive a diagnosis of normal cognitive function, dementia, Mild Cognitive Impairment (MCI), Cognitive Impairment No Dementia (CIND), depression, or psychosis at each study phase.

#### Dementia

The presence/absence of dementia was defined using the Italian version of the Diagnostic and Statistical Manual of Mental Disorders IV (DSM IV-TR) [38]. After syndromic diagnosis, dementia subtypes were defined as follows. Alzheimer’s disease was assessed using the NINCDS/ADRDA criteria for probable, possible and definite diagnosis [39], as well as the guidelines of the European Federation of Neurological Societies [40]. Vascular Dementia was assessed using the NINDS-AIREN criteria, which are considered to be more pertinent for research purposes than the ADDTC criteria, as well as being useful for other care settings [41]. Dementia with Lewy Bodies was assessed using the criteria outlined in the third report of the DLB Consortium [42]. Frontotemporal Dementia was evaluated using the clinical criteria of the Manchester Royal Infirmary group, having been revised and confirmed by HJ Rosen [43].

#### MCI and CIND

Cognitive impairment without dementia was defined as MCI using Petersen’s criteria [44], requiring a subjective memory complaint, impaired performance on objective memory tests, a non-demented status and absence of dependency or need help attributable to cognitive impairment, while might have been present some difficulties (no “zero” score in ADL and IADL scale). When no self-report of cognitive problems was present, the concept of CIND was applied [45]. Subtypes of cognitive impairment were defined as per the description of MCI by the Working Group of the European Consortium on Alzheimer's Disease [46]. The definition of “cognitive impairment” was applied on the basis of neuropsychological and clinical examinations. A cognitive test score was considered “abnormal” when was under the threshold value of normality gathered from normative studies. The neuropsychological tests and the relevant normative studies are reported in the “Assessment methods” section of this article. The presence of two or more tests below the cut off level was considered a criterion for definition of MCI and CIND. In case of clinical instability and serious language disorders no definite cognitive diagnosis was applied. People with depression and psychosis have been so labeled. To define cognitive status of people with hearing and visual problems only the tests which did not specifically involved the impaired function were considered. In case of low literacy, people could be labeled as “normal” or as “no definite diagnosis” taking into consideration the whole assessment.

Few individuals where considered cognitively impaired, although in the presence of a single abnormal result, only whether there was an agreement between the doctor and the neuropsychologist.

#### Depression

A diagnosis of depression was assigned when the clinical evaluation confirmed the presence of at least three of the following criteria: 1) a history of depression, 2) antidepressant therapy, 3) a score of 8 or more out of 15 on the Geriatric Depression Scale [47]*,* and 4) the positive answer to two key questions on depressed mood, which were derived from the CES-D scale and have been used in epidemiological studies [48]. Otherwise the presence of “depressive symptoms” were considered to be “subthreshold depressive symptoms”, as described by the National Institute of Clinical Excellence (NICE) [49].

#### Psychosis

This diagnosis was assigned only if it was present in the available anamnesis. This category was comprehensive of a wide range of conditions (ICD 10: F20 – F29).

In the case of participants with unstable clinical condition or where there was insufficient information, no diagnosis was defined.

### Assessment methods

Geriatric evaluations were based on the medical history of the participant and a complete physical examination, with special attention given to past neurologic events as well as to present neurologic signs and symptoms. The mental status of the participant was informally evaluated by the physician by asking specific questions based on the DSM IV definition of dementia syndrome, as well as evaluating the ability of the participant to report his or her own medical history. Family doctors and relatives were sometimes interviewed to verify specific information.

Neuropsychological assessments addressed several cognitive areas using the applicable instruments, as listed below. Unless otherwise indicated, normative data were derived from a battery standardized for the Italian population [50]. Global cognition was assessed using the Mini Mental State Examination (MMSE) [51]. MMSE was corrected for age and years of education following the normative data published by Magni et al. [52]. Verbal episodic memory was evaluated using the revised version of the Babcock Story Recall Test and the Rey Auditory-Verbal Learning Test [53]. Language was assessed using the Phonemic Verbal Fluency Tests [54] and Semantic Verbal Fluency Test. Executive functions were gauged using Raven’s Coloured Matrices [55] and Clock Drawing Test [56]. Simple and divided attention, and attentional control were tested using the Attentional Matrices and Trail Making Test [57]. Finally, visuospatial skills were evaluated using the Rey-Osterrieth Complex Figure (copy and recall) [58]. Each evaluation session was preceded by an informal interview to evaluate potential interfering factors and to help the participants feel at ease.

Functional evaluations of activities of daily living were assessed using the Katz Activity of Daily Living scale [59]. The Instrumental Activities of Daily Living scale was adopted to investigate eight more complex daily tasks such as the ability to use the telephone, prepare meals or handle finances [60].

A final diagnosis was assigned by the Research Director after reaching an agreement between the neuropsychological and geriatric examinations.

### Social, clinical and lifestyle parameters

We conducted a comprehensive clinical assessment, where we obtained individual medical histories and performed a full physical examination of each participant. The following anthropometric measurements were obtained: height, knee height (the distance between the upper ridge of the patella and the heel) [61], weight (measured to the nearest 100 g), waist (measured at the end of normal expiration at the midway between the lower rib and the iliac crest) and head circumference (measured just above the supraorbital ridges) [62].

We also conducted the Talking While Walking Test in which walking speed was recorded during a single and dual action task [63]. Participants were asked to rise from a chair and to walk back and forth along an indicated distance of 5 meters with no pausing, and to sit down again. They were then asked to perform the same movements while listing personal names in a loud voice (female names for men and male names for women).

Data regarding social and lifestyle factors were collected using a specific questionnaire. First, we gathered demographic information such as gender, age, and education. To assess occupational history, different occupations were categorized into four classes that were derived from the nine classes used by the Italian Statistical Institute [64]. We also recorded the reported levels of physical and mental stress associated with the primary occupation for each participant. To assess socio-economic status, we asked seven questions that probed the past and current economic status, social relationships, hobbies, leisure activities, and interests of each individual. Adverse life events were measured using the "Geriatric Assessment Life Events Scale (GALES)", a scale developed and validated for the geriatric population. The number of events, level of stress, and personal responses to these events were recorded [33]. We also assessed alcohol, coffee, and tea consumption using simple and reliable questions about alcohol consumption, which in Italy is mainly that of wine [65]. To obtain information about smoking habits we included questions about the type (cigarettes, cigar, pipe), daily frequency, and years of smoking. To assess diet we asked 13 questions about perceptions of change in appetite and food consumption, with special attention to fish [66] and fresh vegetables [67]. Physical activity and exercise were measured by evaluating physical activities related to daily needs separately from leisure time activities. The weekly frequency of different physical activities was recorded. Sleep quality was evaluated using a reliable set of questions [68] concerning duration of sleep, insomnia, the use of sleep medication, sleep apnea, and other sleep disorders. In measuring perceived health and perceived age, health was self-rated as “very good”, “good”, “fair”, “poor”, or “very poor”, [69,70] together with any perceived changes in the last year. Perceived physical age and perceived mental age were rated either as feeling younger, the same, or older in comparison with one’s chronological age [32]. Finally, technology use was evaluated by asking questions about use of simple technological instruments such as remote controls, credit cards, and computers [35].

Biological markers were investigated using a blood sample collected from each participant while in a sitting position. Part of the blood sample was used to assess thyroid function (TSH), cyanocobalamin, folate, and blood cell count, while the remaining part was drawn and stored for other biological analyses. Specifically, from a 12 ml EDTA blood sample, 1.5 ml was stored at −80°C for DNA extraction and about 10 ml was centrifuged at 2500 rpm for 15 minutes to obtain plasma that was then stored at −80°C. The remaining blood sample was diluted with phosphate buffered saline (PBS, GIBCO) and the lymphocytes were isolated using a Ficoll-Paque PLUS gradient according to the manufacturer's instructions (GE Healthcare) and stored at −80°C.

## Discussion

**Figure 2:**
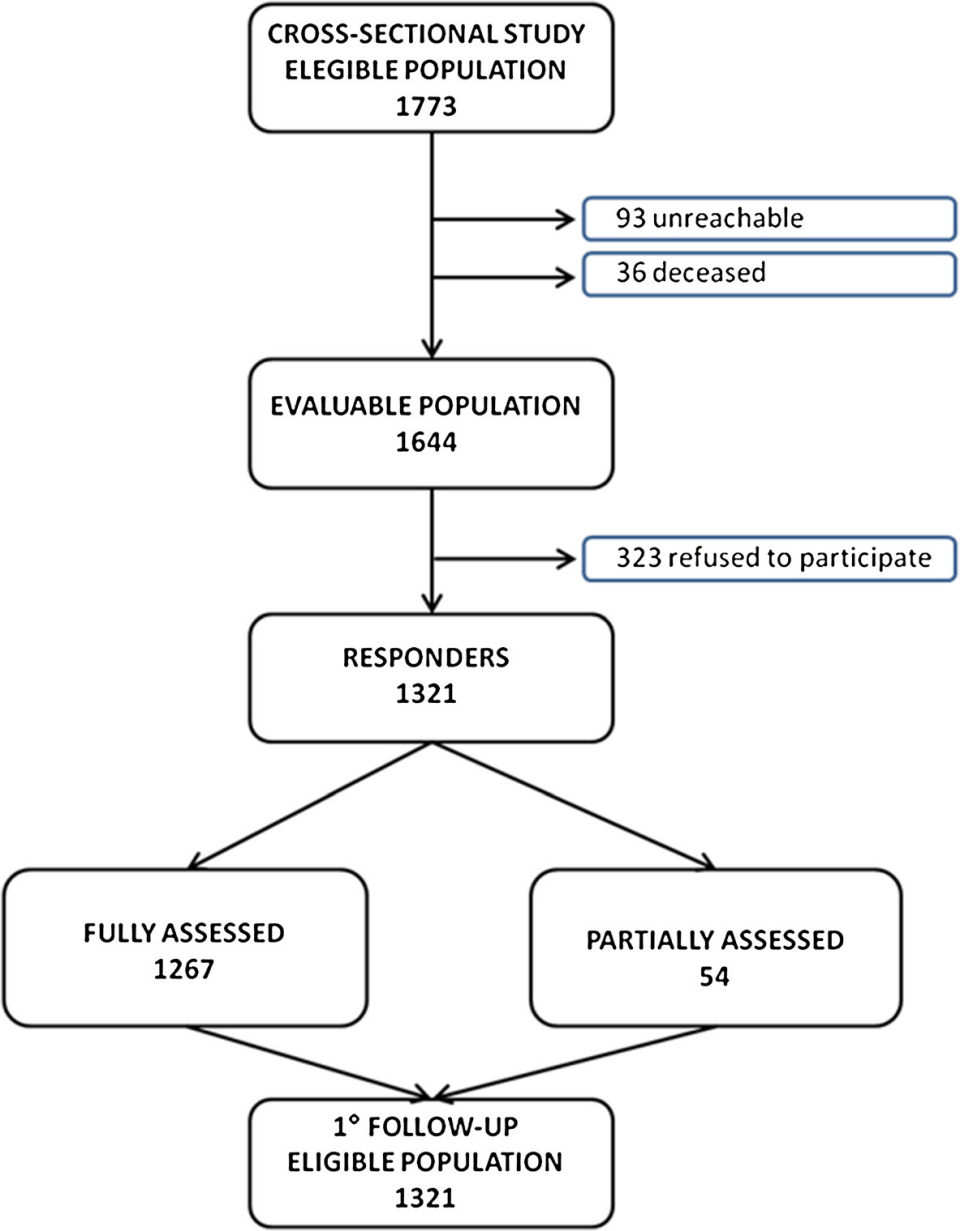
Flow chart of the “InveCe.Ab” study.

The cross-sectional phase of the study was completed at the end of 2010 and a progress flow-chart is given in Figure 2. In detail, the extraction of data about the eligible population from the registry office in November 2009 identified 1773 people; a total 1644 of whom we were able to contact (some had moved away, changed residence, or passed away before we were able to contact them). A total of 1321 people out of the 1664 participated in the cross-sectional study. Our study had a considerable response rate of 80.35%, while other population studies of those over 65 years of age in Italy have had a return rate of 75% at most [71].

This high return rate was likely due to the style of recruitment and personalization of the successive contacts. Among the participants, 543 (47%) volunteered to participate during the initial presentation meetings, 621 (41.1%) agreed to participate after one attempt at personal contact had been made (via a letter and telephone call), and the other 157 (11.9%) participated only after receiving several letters and telephone calls. Among the 1321 participants, 1267 (95.9%) were fully assessed and only 54 (4.1%) were partially assessed. All responders, whether completely or partially assessed, represent the eligible population for the follow up phase of the study.

The prospective investigation of a population can significantly increase understanding of the relationships between age, cognitive decline, and dementia, as well as a wide array of factors associated with healthful cognitive aging. A major challenge in the quest to promote healthful aging is to uncover the determinants of cognitive decline and identify risk factors that lead to dementia.

Adding further evidence to the available data pool regarding the influence of lifestyle and biology on cognitive aging and dementia may help us develop strategies to delay aging-associated cognitive loss; an attainable goal with a considerable public health impact.

### Organizational structure

Our multidisciplinary study group includes diverse professionals (geriatricians, neuropsychologists, biologists, social workers and statisticians) who are able to offer different perspectives regarding the selection of data and interpretation of results.

## Competing interests

The authors declare that they have no competing interests.

## Authors’ contribution

AG, principal investigator, was responsible for the study design and drafted the manuscript; MC and SFV conceived the medical section and helped to draft the manuscript.; RV conceived and carried out the neuropsychological evaluations; SF planned the execution of the study and helped to draft the manuscript; GF participated in the design of the study; LP and AD carried out the collection of blood samples, plasma separation and lymphocytes extraction and helped to draft the manuscript; VVF and SV participated in the design of the study, planned epidemiological and statistical design and helped to draft the manuscript. All co-authors reviewed the manuscript and approved the final manuscript.

## Pre-publication history

The pre-publication history for this paper can be accessed here:

http://www.biomedcentral.com/1471-2318/13/98/prepub
